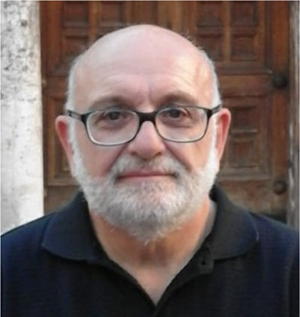# In memoriam Dr. Josep María Queraltó, destacado miembro de la SEQC^M^ ^L^ desde hace 35 años

**DOI:** 10.1515/almed-2020-0075

**Published:** 2020-09-22

**Authors:** Junta Directiva de la Sociedad Española de Medicina del Laboratorio

La Sociedad Española de Medicina de Laboratorio (SEQC^ML^) lamenta tener que comunicar el fallecimiento de uno de sus más ilustres socios, el Dr. Josep Maria Queraltó, de 67 años de edad, quien nos abandonó el pasado viernes, 27 de marzo, de forma repentina en su domicilio de Sant Andreu de Llavaneres (Barcelona).

El Dr. Queraltó ha sido un miembro muy implicado en la Sociedad Española de Medicina de Laboratorio durante 35 años. Entre sus actividades a nivel nacional destacan su puesto como secretario de la Junta Directiva de esta Sociedad entre 1990 y 1996, la presidencia de la antigua Comisión de Valores de Referencia y del Comité de Educación durante los años 80 y 90 y la presidencia de la Comisión de Monitorización de Fármacos y Toxicología Clínica durante dos periodos (2004–2005 y 2013–2018).

Además, fue miembro de la Comisión de Valor Semiológico de las magnitudes Bioquímicas (1985–1989) y del Comité Científico de esta Sociedad (1985–1992), así como del Comité Editorial de la Revista Avances en Medicina de Laboratorio y del Consejo de Dirección; puestos, estos dos últimos, que conservaba en la actualidad.

Asimismo, ha sido de gran importancia su labor representando a la SEQC^ML^ en organismos internacionales, donde ha ocupado puestos de gran trascendencia, como la presidencia de la junta de la Federación Europea de Sociedades Nacionales de Química Clínica (FESCC). También ha sido miembro del Comité del Registro Europeo EC4 desde el año 2000 hasta la actualidad.

Su perseverancia en las diferentes tareas internacionales en las que ha participado y los contactos internacionales, que cuidó de forma exquisita, han favorecido que la SEQC^ML^ haya estado presente en numerosos eventos internacionales entre los que cabe destacar, por su gran impacto, la organización de los Congresos EuroMedLab Barcelona 2003, del cual fue presidente del Comité Científico, y EuroMedLab Barcelona 2019, del cual fue miembro del Comité Organizador. El éxito de ambos Congresos Europeos ha dado un enorme impulso y proyección de la SEQC^ML^ a nivel internacional.

En los días posteriores a su deceso, la Sociedad ha recibido decenas de correos electrónicos y manifestaciones escritas de diferentes países del mundo en los que se manifestaba el dolor y la consternación por la pérdida de una persona tan querida y valorada. Dentro de su gran valía humana y profesional, todos los que tuvieron la oportunidad de tratar con él destacan su paciencia y su gran visión de futuro en el campo de la formación en Medicina de Laboratorio.

**Figure:**
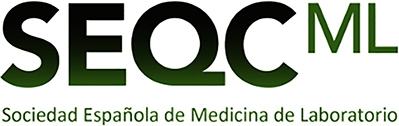

**Figure:**